# A Provegetarian Food Pattern Emphasizing Preference for Healthy Plant-Derived Foods Reduces the Risk of Overweight/Obesity in the SUN Cohort

**DOI:** 10.3390/nu11071553

**Published:** 2019-07-09

**Authors:** Clara Gómez-Donoso, Miguel Ángel Martínez-González, J. Alfredo Martínez, Alfredo Gea, Julen Sanz-Serrano, Federico J. A. Perez-Cueto, Maira Bes-Rastrollo

**Affiliations:** 1 Department of Preventive Medicine and Public Health, School of Medicine, University of Navarra, 31008 Pamplona, Spain; 2 Biomedical Research Centre Network of Physiopathology of Obesity and Nutrition (CIBERobn), Institute of Health Carlos III, 28029 Madrid, Spain; 3 Navarra Institute for Health Research (IdiSNA), 31008 Pamplona, Spain; 4 Department of Nutrition, Harvard TH Chan School of Public Health, Boston, MA 02115, USA; 5 Department of Nutrition, Food Sciences and Physiology, School of Pharmacy and Nutrition, University of Navarra, 31008 Pamplona, Spain; 6 Department of Pharmacology and Toxicology, School of Pharmacy and Nutrition, University of Navarra, 31008 Pamplona, Spain; 7 Food Design and Consumer Behavior Section, Department of Food Science, University of Copenhagen, Rolighedsvej 26, 1958 Frederiksberg C, Denmark

**Keywords:** overweight, obesity, dietary patterns, vegetarian, provegetarian, epidemiology, nutrition, prospective cohort study

## Abstract

Provegetarian diets (i.e., preference for plant-derived foods but not exclusion of animal foods) have been associated with a reduced risk of long-term weight gain and could be more easily embraced than strict vegetarian diets. However, not all plant-derived foods are equally healthy. In the “Seguimiento Universidad de Navarra” (SUN) cohort, we prospectively evaluated the association between different provegetarian food patterns and the incidence of overweight/obesity in 11,554 participants with initial body mass index <25 kg/m^2^. A provegetarian food pattern (FP) was built by assigning positive scores to plant foods and reverse scores to animal foods. A healthful and an unhealthful provegetarian FP, which distinguished between healthy (fruits/vegetables/whole grains/nuts/legumes/olive oil/coffee) and less-healthy plant foods (fruit juices/potatoes/refined grains/pastries/sugary beverages), were also built. A total of 2320 new cases of overweight or obesity were identified after a median follow-up of 10.3 years. Higher baseline conformity with the overall provegetarian FP was inversely associated with overweight/obesity (HR comparing extreme quintiles: 0.85; 95% CI: 0.75 to 0.96; p-trend: 0.014). This association was stronger for the healthful FP (HR: 0.78; 95% CI: 0.67 to 0.90; p-trend: <0.001) and was not apparent for the unhealthful FP (HR: 1.07; 95% CI: 0.92 to 1.23; p-trend: 0.551). In a large prospective cohort of relatively young adults, better conformity with a healthy provegetarian diet was associated with a reduced long-term risk of overweight/obesity, whereas no consistent trend was found for a FP that emphasized less-healthy plant foods.

## 1. Introduction

It is well-established that diets higher in plant-based foods and with fewer animal source foods confer both health and environmental benefits [1,2,3]. Plant-based diets have been associated with a lower risk of various diseases [4,5,6,7,8,9,10,11], including a lower risk of obesity [12,13,14,15,16,17,18,19], which is among the greatest contributors to premature mortality [20,21,22]. Moreover, whereas strict vegan or vegetarian diets might not be easily embraced within the general population, provegetarian diets (i.e., preferentially but not exclusively consuming plant-derived foods) based on gradual dietary changes may be easier to adopt. Although this type of plant-based diet is also commonly known as flexitarian, we used the provegetarian operational score because it has been defined in a quantitative and easily reproducible way [10]. However, with the exception of several recent studies [23,24,25,26,27], prior studies of plant-based diets do not take into account the fact that not all plant-derived foods are equally healthy.

Since the potential mechanisms responsible for the beneficial effects of plant-based diets on body weight [28,29,30,31,32,33,34,35,36,37] are mainly supported by dietary constituents like fiber and other biologically active micronutrients which are characteristic of healthy plant foods (i.e., fruits and vegetables), it is warranted to evaluate whether the healthfulness of plant-derived foods influences the risk of long-term weight gain. Additionally, it is important to provide evidence towards the advantages and disadvantages of strategies that consumers may put in practice when they adopt plant-based diets following the societal trends, and to provide insights for tomorrow’s food innovation. Therefore, we aimed to prospectively examine the association between different versions of a provegetarian food pattern and the incidence of overweigh/obesity in the SUN (Seguimiento Universidad de Navarra/University of Navarra Follow-up) cohort.

## 2. Materials and Methods

### 2.1. Study Population

The “Seguimiento Universidad de Navarra” (SUN) prospective cohort study started on December 1999 following the models of the Nurses’ Health Study and the Health Professionals Follow-Up Study. It is a multipurpose and dynamic cohort (i.e., recruitment is permanently open) composed of young middle-aged university graduates from different Spanish universities, including the University of Navarra. Baseline assessment and follow-up information is gathered through postal or web-based questionnaires completed by participants every 2 years. Self-administered questionnaires include information on sociodemographic, lifestyle and dietary variables as well as the use of medication and the prevalence or incidence of diseases during follow-up. For the main outcomes, medical records are requested to participants or their next of kin in order to confirm the diagnoses by a panel of medical doctors of the study, blinded to the dietary habits of participants and other exposures of interest. For participants lost to follow-up, the National Death Index is periodically checked to determine their vital status and cause of death. The overall retention in the cohort is higher than 90%. All procedures were approved by the Institutional Review Board of the University of Navarra (2017.151) according to Declaration of Helsinki guidelines. Voluntary completion of the baseline self-administrated questionnaire was considered to imply informed consent, as participants received detailed information about the study. Further details about the methodology and characteristics of participants have been described in more detail elsewhere [38].

**Figure 1:**
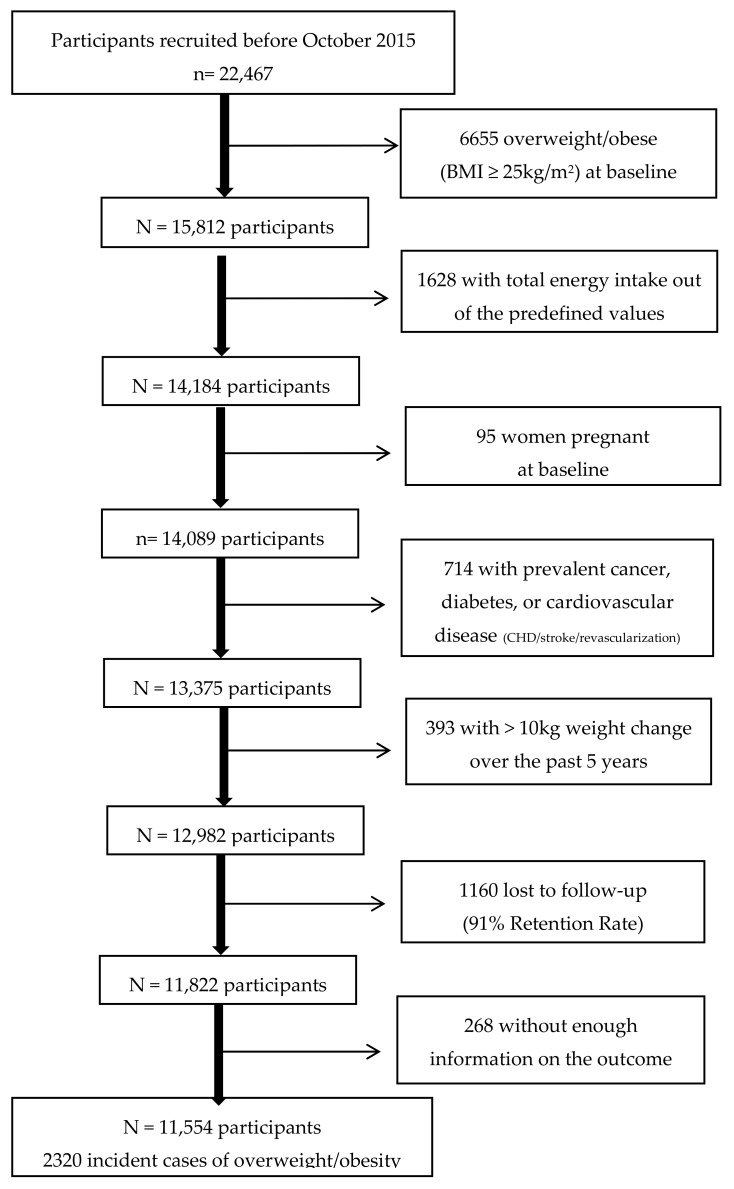
Flow chart depicting the selection process among participants of the Seguimiento Universidad de Navarra (SUN) cohort.

Up to July 2018, there were 22,791 participants who had completed the baseline questionnaire. In order to ensure that all participants had completed at least one follow-up questionnaire, only those who were recruited before October 2015 were considered eligible. Out of the 22,467 eligible participants, we excluded 6655 with prevalent overweight or obesity (body mass index (BMI) ≥ 25 kg/m^2^) at baseline, 1628 who reported implausible values for total energy intake according to predefined limits (>4000 kcal/d in men and >3500 kcal/d in women or <800 kcal/d in men and <500 kcal/d in women), 95 women who were pregnant at baseline, 714 with prevalent chronic disease (diabetes, cardiovascular disease and/or cancer), and 393 with a weight change > 10 kg in the 5 years before entering the study to reduce the potential source of confounding by other causes of weight changes. Among the remaining 12,982 participants, 1160 were lost to follow-up leaving a total of 11,822 (retention = 91%). Additionally, we excluded 268 participants who had missing values in the variables of interest regarding the outcome. After exclusions, a sample of 11,554 participants was included in this study (Figure 1).

### 2.2. Dietary Assessment

A 136-item semi-quantitative food frequency questionnaire (FFQ) repeatedly validated in Spanish participants [39,40] was completed at baseline. Frequencies of consumption were measured in 9 categories (ranging from never/almost never to >6 servings/day) for each food item, in order to assess typical food intake over the previous year. Daily intake (grams/day) was calculated by multiplying the specified portion size for each food item by the frequency of consumption. Nutrient intake was updated by a trained dietician using the latest available information on Spanish food composition tables [41,42]. The scoring criteria for the overall, healthful and unhealthful provegetarian food patterns (FPs) are showed in Table 1, as described in previous studies [10,23]. Information on the food items from the FFQ included in each food group is shown in Table S1. Differences in food consumption between the lowest and highest quintiles of the provegetarian FPs are graphically represented in Figures S1–S3, showing differences (in percentage) between medians of extreme quintiles (quintile 1 and 5) and the median of the whole sample.

Adherence to an overall provegetarian FP was calculated according to the score proposed by Martínez-González et al. [10], which quantifies the habit of preferentially consuming plant-derived foods instead of animal-derived foods without the need to follow a strict vegetarian diet. This score quantifies the energy-adjusted consumption (g/d) of seven plant food groups (fruits, vegetables, potatoes, nuts, legumes, cereal grains and olive oil) and five animal food groups (dairy, eggs, meat, fish and seafood and animal fat). The residual method was used separately for men and women to adjust for total energy intake and the energy-adjusted estimates (residuals) were ranked according to their sex-specific quintiles. The quintile values for animal food groups were reversed (a value of 5 was assigned for the first quintile, 4 for the second quintile, and successively until the value of 1 was assigned to the fifth quintile). To obtain the overall provegetarian FP score, quintile values of plant foods and reverse quintile values of animal foods were summed. Thus, the final scores assigned to participants could range from 12 (lowest adherence) to 60 (highest adherence).

Likewise, adherence to a healthful and unhealthful provegetarian FP was calculated according to the scoring criteria suggested by Satija et al. [23]. Healthy (fruits, vegetables, whole grains, nuts, legumes, olive oil, coffee) and less-healthy plant foods (fruit juices, potatoes, refined grains, pastries, sugary beverages) were distinguished. As shown in Table 1, to build the healthful provegetarian FP, positive scores were assigned to healthy plant foods and reverse scores to less-healthy plant foods as well as to animal foods. For the unhealthful provegetarian FP, positive scores were assigned to less-healthy plant foods and reverse scores to healthy plant foods and animal foods. Quintiles and reverse quintiles were summed to obtain scores of the healthful and unhealthful versions of a provegetarian FP. Participants were categorized into energy-adjusted quintiles of the healthful and unhealthful provegetarian FPs. Thus, their final scores could range from 18 (lowest adherence) to 90 (highest adherence).

Adherence to the Mediterranean diet was assessed using the a priori 9-item Mediterranean diet score (MDS) proposed by Trichopoulou et al. [43].

### 2.3. Other Covariates Assessment

The baseline questionnaire gathered information about sociodemographic and anthropometric characteristics, health-related habits, and clinical data including personal and family history of disease. Physical activity was assessed using a previously validated questionnaire, which included 17 different activities during leisure time [44]. Metabolic equivalents (METs) were calculated to yield MET-hours per week scores for each participant.

Baseline dietary intake data derived from food composition tables for Spain [41,42] were used to identify the fat quality index (FQI) and the carbohydrate quality index (CQI). The FQI was calculated using the ratio (monounsaturated + polyunsaturated fatty acids)/(saturated + trans fatty acids) as a continuous variable, ranging from 1.01 to 4.93. The CQI was determined by summing up quintiles of the following four criteria: Dietary fiber intake (g/day), glycemic index, the ratio whole grains/total grains and the ratio solid carbohydrates/solid + liquid carbohydrates. All criteria had the same weighting, and the CQI ranged from 4 to 20. The FQI and CQI have been previously used in this cohort [45] and also in the Prevención con Dieta Mediterránea (PREDIMED) study [46].

### 2.4. Outcome Ascertainment

Weight of participants was self-reported at baseline and every 2 years in the follow-up questionnaires. BMI was calculated as the self-reported weight in kilograms divided by the square of height in meters. Weight was self-reported by participants at baseline and every 2 years of follow-up. BMI was calculated as weight in kilograms divided by the square of height in meters, which was ascertained at baseline. Anthropometric data (i.e., self-reported weight and BMI) have been previously validated in a subsample of this cohort finding strong correlation results [47]. The outcomes were: (1) Incidence of overweight/obesity (BMI ≥ 25 kg/m^2^); and (2) average change in body weight (grams) per year (g/y).

### 2.5. Statistical Analysis

Inverse probability weighting was used to determine the age- and sex-adjusted baseline characteristics of participants according to quintiles of adherence to the different provegetarian FPs in order to remove differences that were only explained by the different age and sex distribution across baseline quintiles of provegetarian FPs.

Cox proportional hazards analyses were conducted to assess the relationship between provegetarian FPs with varying degree of healthfulness and the incidence of overweight/obesity. All models included age as underlying time variable and were stratified by age groups and year of recruitment. After crude analyses, a model adjusted for sex and age was fitted. Multivariable models were additionally adjusted for known risk factors of weight gain and potential confounders by using baseline values of the following covariates: Baseline BMI (kg/m^2^, continuous), physical activity (METs-h/week, quartiles), hours of TV watching (quartiles), smoking status (never, current, or former), marital status (single, married, other), years of university education (continuous), total energy intake (kcal/day, continuous), snacking between meals (yes, no), following a special diet at baseline (yes, no), parental family history of obesity (yes, no) and hours of siesta sleep (0, >0 to ≤0.5, >0.5). Robust standard errors were used. In order to reduce the effects of potential measurement errors due to diet variation during follow-up, dietary data were updated after 10 years of follow-up if the participant was followed for longer than 10 years and had completed the 10-year follow-up FFQ. Tests of linear trends were conducted using the medians of each provegetarian score for each quintile and treating the variable as continuous in Cox models. Moreover, to minimize the potential distortions produced by different measurement units in each provegetarian score, z scores were used. Each z score was calculated for each participant as the score value of the participant minus the mean value for the overall sample divided by the standard deviation of the score in the overall sample.

Multiple linear regression models were used to assess the association between the provegetarian FPs and the average yearly weight change during follow-up, considering the first quintile of adherence as the reference group. We estimated β regression coefficients (and their 95% confidence interval (CI)) for the other four quintiles, which should be interpreted as the difference in average yearly weight change (g/y) for each of the upper four quintiles versus the lowest quintile. To take into account repeated measurements of diet, generalized estimating equations with an unstructured correlation matrix were used to assess the relationship between updated scores of the provegetarian FPs and average yearly weight change. Analyses were adjusted for the same confounding factors as the main Cox regression analysis.

Additional analyses were conducted after excluding participants with a baseline BMI ≥ 30 kg/m^2^ instead of BMI ≥ 25 kg/m^2^ (as in the main analyses, which only included normal weight participants) in order to assess the incidence of obesity among overweight participants (i.e., change of BMI category from overweight to obese).

Sensitivity analyses were conducted to explore the robustness of our findings by rerunning the analyses under the following scenarios: Excluding participants with energy intake between the 5th and 95th percentiles; excluding participants with no answer in > 12 items in the 136-item baseline FFQ; including participants with weight change > 10 kg over the previous 5 years before entering the study; excluding participants with BMI > 24.5 kg/m^2^ at baseline (even though self-reported BMI has been previously validated finding good correlation results, some degree of misclassification might still exist); excluding early incident cases of overweight (participants who became overweight or obese only during the first 2 y of follow-up); additionally adjusting for weight gain ≥ 3 kg over the previous 5 years before entering the cohort; considering as the outcome only obesity (BMI ≥ 30 kg/m^2^) instead of overweight (BMI ≥ 25 kg/m^2^) and truncating follow-up at 10 years.

All analyses were performed using STATA/SE version 12.0 (StataCorp, College Station, TX, USA).

## 3. Results

A total of 11,554 participants (8419 women and 3135 men; 34.7 ± 10.8 years [mean age ± SD]) were followed for a median of 10.3 years. Age-and sex-adjusted baseline characteristics of participants according to quintiles of adherence to the different provegetarian FPs are presented in Tables S2–S4. Compared with participants in the first quintile, those in the fifth quintile with higher scores on the overall and healthful provegetarian FPs were more likely to live alone, follow a special diet, be non-smokers, be more physically active, have a family history of obesity, and have a higher total energy and dietary fiber intake. Conversely, those with high scores on the unhealthful provegetarian FP were less physically active, less likely to live alone, more likely to smoke, have snacks, have a higher total energy intake and lower total fiber intake than those with low scores.

**Figure 2:**
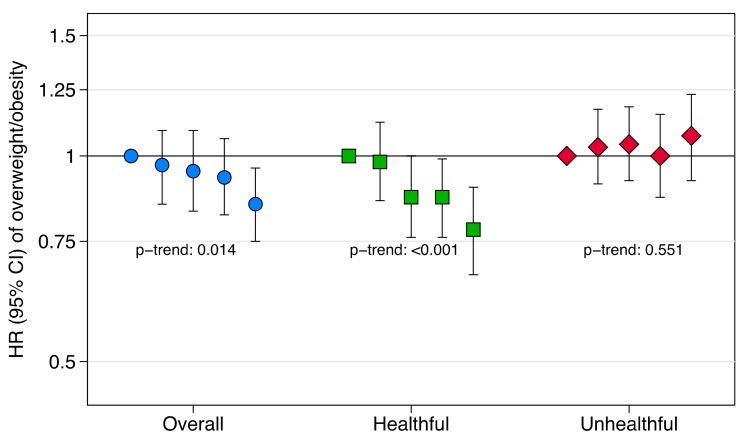
Hazard ratios (HR) and 95% confidence intervals (CI) of overweight/obesity according to baseline quintiles of the provegetarian food patterns.

Over 113,213 person-years of follow-up, a total of 2320 new cases of overweight/obesity (BMI ≥ 25 kg/m^2^) were identified. Hazard ratios (HR) and their 95% confidence intervals (CI) for the risk of overweight/obesity in the age-and sex-adjusted and multivariable-adjusted models are shown in Table 2. In the fully adjusted models, the overall provegetarian FP was inversely associated with overweight/obesity incidence (HR comparing extreme quintiles: 0.85; 95% CI: 0.75 to 0.96; HR for each additional 2 z-score units: 0.89; 95% CI: 0.82 to 0.97; p-trend = 0.014). When we distinguished among healthful and unhealthful provegetarian FPs, we found a stronger inverse association between the healthful provegetarian FP and overweight/obesity incidence (HR comparing extreme quintiles: 0.78; 95% CI: 0.67 to 0.90; HR for each additional 2 z-score units: 0.84; 95% CI: 0.77 to 0.92; p-trend < 0.001), whereas null findings were observed for the unhealthful provegetarian FP (HR comparing extreme quintiles: 1.07; 95% CI: 0.92 to 1.23; HR for each additional 2 z-score units: 1.00; 95% CI: 0.91 to 1.10; p-trend = 0.551). Results from the multivariable-adjusted models are also graphically represented in Figure 2, showing HRs and 95% CIs of overweight/obesity according to baseline quintiles of the 3 provegetarian FPs.

Consistently, absolute average yearly weight change (g/y) modestly decreased across quintiles of overall and healthful provegetarian FPs (i.e., higher adherence to both overall and healthful provegetarian FPs was associated with less weight gain over time) and remained fairly constant across quintiles of the unhealthful provegetarian FP (Table 3). The average difference in yearly weight gain between extreme quintiles of adherence to the overall provegetarian FP was −167 g (95% CI: −249 to −84) and for the healthful provegetarian FP it was −202 g (95% CI: −294 to −110).

In an additional analysis conducted to assess the effect of several provegetarian FPs on incidence of obesity among overweight participants, a total of 669 new cases of obesity (BMI ≥ 30 kg/m^2^) were identified. HRs and their 95% CIs for the risk of obesity in the age-and sex-adjusted and multivariable-adjusted models are shown in Table 4. Fully adjusted estimates suggested that the inverse association between the overall and healthful provegetarian food patterns and overweight/obesity among normal weight participants is not as evident among initially overweight subjects (HR comparing extreme quintiles of the overall provegetarian food pattern: 0.79; 95% CI: 0.61 to 1.02; p-trend = 0.056. HR comparing extreme quintiles of the healthful provegetarian food pattern: 0.79; 95% CI: 0.59 to 1.05; p-trend = 0.066).

In ancillary analyses (data not shown), we investigated the association between adherence to an unhealthful provegetarian FP exclusively composed of plant-based foods (less-healthy plant foods positively weighted and healthy plant foods negatively weighted) and risk of overweight/obesity. We also explored the association between an unhealthful provegetarian FP composed of less-healthy plant foods (positively weighted) and animal foods (negatively weighted) and risk of overweight/obesity. The purpose of running these ancillary analyses was to study the independent effects of consuming more less-healthy plant foods compared to healthier plant foods (HR comparing extreme quintiles: 1.21; 95% CI: 1.05 to 1.40; p-trend: (0.021) and compared to more animal foods (HR comparing extreme quintiles: 0.91; 95% CI: 0.80 to 1.05; p-trend: (0.130).

**Figure 3:**
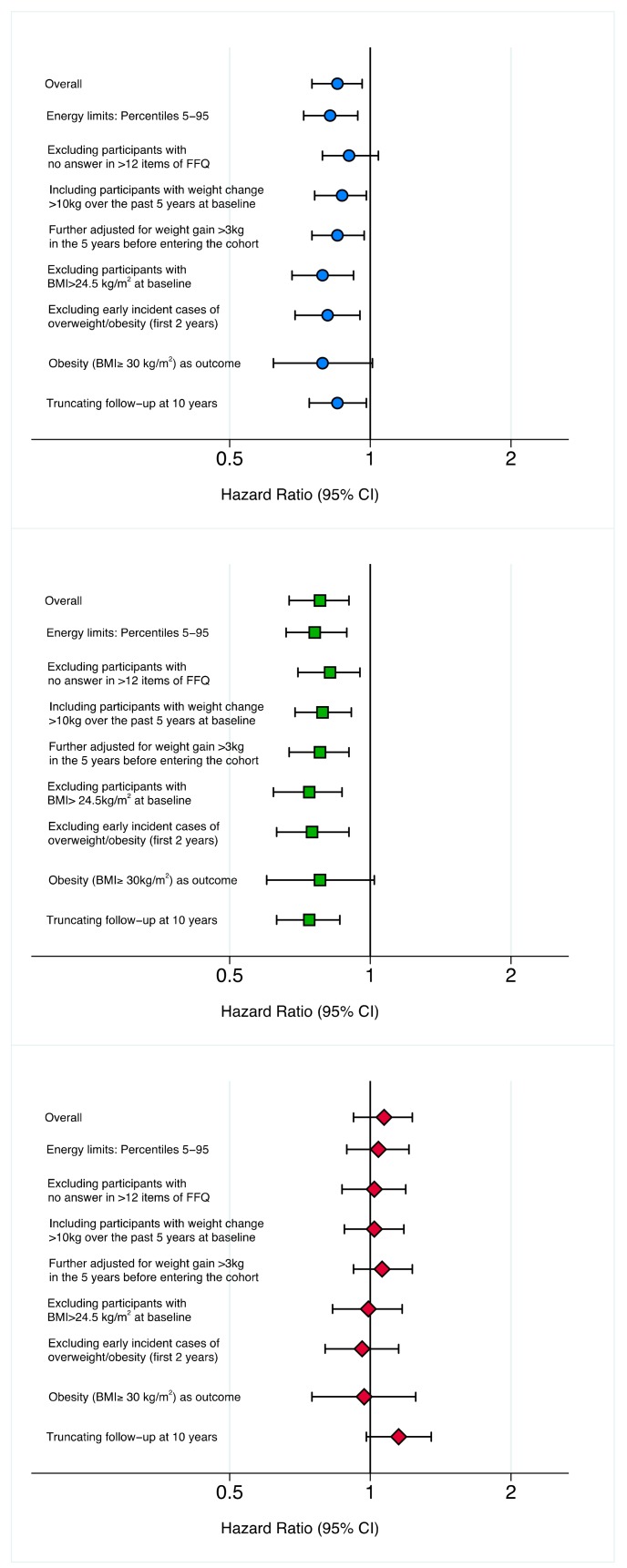
Sensitivity Analyses (results under different scenarios). Hazard Ratios (95% CI) of incident overweight/obesity for extreme quintiles (Q5 vs. Q1) of adherence to the overall, healthful and unhealthful provegetarian food patterns.

Sensitivity analyses were conducted to assess the robustness of our results under a variety of scenarios (Figure 3 and Table S5). The associations of overall and healthful provegetarian FPs with the risk of overweight/obesity were attenuated when participants with no answer in >12 out of the 136 items included in the baseline FFQ were excluded and when obesity (BMI ≥ 30 kg/m^2^) was the main outcome considered. Slightly stronger associations were found when participants with baseline BMI > 24.5 kg/m^2^ were excluded. The adverse association with the unhealthful provegetarian FP became stronger when the follow-up period was truncated at 10 years.

## 4. Discussion

In a Mediterranean dynamic prospective cohort study composed of university graduates with an initial BMI < 25 kg/m^2^ (normal weight), we found that a general preference for plant-derived foods (provegetarian FP) was modestly associated with lower risk of subsequently developing overweight/obesity during follow-up. This inverse association was considerably stronger when we specifically evaluated the preference for healthy plant-derived foods (healthful provegetarian FP), whereas no such association was found for the preference for less healthy plant-derived foods (unhealthful provegetarian FP). Our findings were robust in sensitivity analyses and remained consistent and significant when updating diet measurements with the use of repeated nutritional data obtained from the FFQ administered after 10 years of follow-up. Additional analyses including normal weight and overweight participants with an initial BMI < 30 kg/m^2^ revealed that we cannot be equally certain about the inverse association between these provegetarian food patterns and obesity among overweight subjects. These results are in line with recent findings from three ongoing prospective cohort studies in the United States, which were published while our paper was under review [25].

A-priori defined provegetarian indices were used to assess the effect of preference for plant-derived foods compared with preferential selection of foods from animal sources on the risk of new-onset overweight/obesity. This approach builds on previous research and includes well-defined food groups with varying nutritional content. Thus, results are in close agreement with previous findings, which could be expected, as most healthy plant foods positively weighed in the healthful provegetarian FP (vegetables, fruits, legumes, whole grains, nuts, olive oil, coffee) have been consistently associated with lower risk of weight gain. Likewise, less-healthy plant foods positively weighed in the unhealthful provegetarian FP (fruit juices, potatoes, refined grains, sugary beverages, pastries) have been consistently associated with higher risk of weight gain [48]. Noteworthy, most of the cereal grains consumed by our participants were refined, especially white bread, which accounted for 49% of the cereal group. In addition, according to the NOVA (a name, not an acronym) food classification system that categorizes foods based on the extent and purpose of food processing, plant-based foods such as sugary beverages and pastries can be considered ultra-processed foods. Their convenience (imperishable, ready-to-consume), hyper-palatability, branding and aggressive marketing give ultra-processed foods enormous market advantages over minimally processed healthy food, and their consumption has been systematically associated with the deterioration of overall nutritional quality of diets and with several chronic non-communicable diseases including obesity [49]. Therefore, from a public health perspective, focusing solely on plant or animal origin of foods may not be the most effective message in terms of obesity prevention.

The associations observed between a general preference for plant-derived foods and lower risk of overweight/obesity are consistent with previous research [12,13,14,15,16,17,18,19,20]. In particular, preference for less-healthy plant foods was associated with an increased risk of overweight/obesity compared with preferential selection of healthier plant foods, as could be expected. However, preference for less-healthy plant foods did not affect the risk of overweight/obesity compared with preference for foods from animal sources. These results suggest that plant-based foods should be prioritized over animal foods, and reinforce the importance of a preference for healthier plant foods in terms of weight gain prevention. Similarly, a recent study found that exclusion of less healthy plant-based foods from a plant-based diet index did not substantially change its beneficial effect, while exclusion of the healthy plant-based foods combined from the plant-based diet index moderately attenuated the inverse association. Therefore, it seems that healthy plant foods could apparently be driving the observed beneficial effect. The associations were also moderately attenuated by giving healthier animal foods positive scores [12]. Additionally, another study found that a greater consumption of sugar-sweetened beverages and ultra-processed food was associated with a higher risk of being overweight among vegetarians [50], which further supports our results. Hence, an additional implication of this study is to call for evidence regarding the effects on health and sustainability of innovatively processed plant-based products that are becoming increasingly common among consumers.

The potential mechanisms underlying our findings may be related to the preventive effect against overweight/obesity of a high intake of foods containing dietary fiber and bioactive compounds, which are naturally found in fruits and vegetables. Minimally processed plant-based foods are typically low in energy density and contain high concentrations of micronutrients and phytochemicals like flavonoids that may have a beneficial influence on energy homeostatic pathways [51]. Other components like fiber may also increase satiation and positively modulate gut microbiota composition [30]. In fact, we found evidence that fiber may mediate the beneficial effect of a provegetarian diet on overweight/obesity (i.e., the inverse association was attenuated after additionally adjusting for total fiber intake [g/day]). Another study in the SUN cohort found a significant inverse association between the CQI and the incidence of overweight/obesity, which highlights the importance of preferentially consuming high-quality carbohydrates in terms of obesity prevention [52]. Moreover, vegetable-and fruit-based dietary patterns have been suggested to modulate inflammation status [53], and a previous study in our cohort found that a higher proinflammatory diet was associated with a higher risk of overweight and obesity [54].

Beyond the health of individuals, and taking into consideration the health of our planet, it is also known that the adoption of provegetarian food patterns could significantly reduce greenhouse gas emissions [1] as well as other environmental impacts, and it is not as expensive as adopting other healthy and environmentally friendly dietary patterns such as the Mediterranean Diet (MedDiet) [55]. This focus may be a useful strategy to change dietary behaviors in the general public towards a healthier diet, as it is a way to connect with deeper needs and desires of people while improving their health as a side effect [56]. Although the MedDiet is also associated with a significantly lower risk of overweight/obesity in our cohort (HR [95% CI] for the highest category of adherence to the MDS [range 6–9] vs the lowest [range 0–3]: 0.78 [0.69 to 0.87]) and represents a food pattern rich in plant-derived foods, there are some important differences between the provegetarian food pattern and the MedDiet in relation to the scoring criteria for fish, potatoes and alcohol. In fact, the provegetarian FP and its healthful version were only moderately correlated (Pearson correlation coefficient: 0.54) with the traditional MedDiet (as defined by the MDS score proposed by Trichopoulou et al. [43]), reflecting that these are novel diet indices that capture unique aspects of a pro-vegetarian diet. Importantly, linking obesity with other major global challenges like climate change and health of the planet focuses attention on the scale and urgency of addressing these challenges and may increase demand for policy action by the public [57]. Our study reinforces current evidence and recommendations that suggest a shift towards diets rich in plant foods with lower intake of animal foods, and emphasizes the importance of prioritizing healthy plant foods. Finally, the present study also provides insights for the development of innovative foods of plant origin by food scientists and the food industry. The design of new foods should be performed within boundaries of key nutrients (e.g., added sugar and salt), but also boundaries in the number of ingredients and their level of processing.

The strengths of the present study include the prospective longitudinal design with a long follow-up period and a relatively large sample size; the restriction to participants with baseline BMI < 25 kg/m^2^, which minimized the possibility of reverse causation bias; and the use of repeated measurements of the diet, which reduced potential measurement errors and increase validity of well-validated self-reported comprehensive food-frequency methods such as the SUN cohort FFQ that has been repeatedly validated in Spanish participants [36,37].

The present study also has some limitations. Although we adjusted for a wide array of potential confounders, we cannot exclude the possibility of residual confounding given the observational nature of the study design and the fact that adherence to the overall and healthful provegetarian was higher among participants with healthier characteristics (non-smokers, higher levels of physical activity). However, the restriction to highly educated participants was applied to control for confounding by socioeconomic status, which is an approach considered in epidemiology as an excellent technique to reduce confounding [58]. This also implies that our sample from the SUN cohort is not representative of the general Spanish population. However, most cohorts are usually non-representative, and “it is not representativeness of the study subjects that enhances the generalization, it is knowledge of specific conditions and an understanding of mechanism that makes for a proper generalization” [59]. Therefore, the dose-response trend found in our results, as well as the consistency with previous studies and the existence of biological plausibility confer high potential of causality for the benefits of a plant-based dietary pattern, particularly a healthy provegetarian pattern, in the prevention of long-term weight gain. Another potential limitation is that weight and dietary information were self-reported so, although they were validated as previously mentioned, we cannot rule out some degree of nondifferential misclassification. Moreover, whole grain consumption has only recently started to be more common in Spain, so the only whole grain product included in the FFQ was brown bread. Therefore, we could not fully consider the effect of a higher consumption of other whole grains on the risk of incident overweight and obesity.

In conclusion, higher adherence to a provegetarian FP emphasizing preference for healthy plant-derived foods was associated with a lower risk of developing overweight and obesity in a cohort of Spanish university graduates with initial low body mass index. Given that excess weight is also a critical risk factor for developing other increasingly frequent non-communicable chronic diseases, a generalized adoption of healthful provegetarian diets is an important target that should be considered in future public health policies.

## Figures and Tables

**Table 1:** Scoring criteria for the provegetarian food patterns.

Provegetarian Food Pattern (Potential Range of 12–60)		Healthful/Unhealthful Provegetarian Food Patterns (Potential Range of 18–90)		
Component	Criteria	Component	Criteria	
Plant food Groups	Energy-Adjusted Quintiles	Plant Food Groups	Energy-Adjusted Quintiles	
		***Healthy***	Healthful	Unhealthful
1. Vegetables	Positive	1. Vegetables	Positive	Reverse
2. Fruits	Positive	2. Fruits	Positive	Reverse
3. Legumes	Positive	3. Legumes	Positive	Reverse
4. Cereal grains	Positive	4. Whole grains	Positive	Reverse
5. Potatoes	Positive	5. Nuts	Positive	Reverse
6. Nuts	Positive	6. Olive oil	Positive	Reverse
7. Olive oil	Positive	7. Coffee	Positive	Reverse
		***Less-healthy***		
**Animal Food Groups**		8. Fruit juices	Reverse	Positive
8. Dairy	Reverse	9. Potatoes	Reverse	Positive
9. Eggs	Reverse	10. Refined grains	Reverse	Positive
10. Meat	Reverse	11. Sugary beverages	Reverse	Positive
11. Fish & seafood	Reverse	12. Pastries	Reverse	Positive
12. Animal fat	Reverse	**Animal Food Groups**		
		13. Dairy	Reverse	Reverse
		14. Eggs	Reverse	Reverse
		15. Meat	Reverse	Reverse
		16. Fish & seafood	Reverse	Reverse
		17. Miscellaneous food	Reverse	Reverse
		18. Animal fat	Reverse	Reverse

**Table 2:** Risk of overweight/obesity (HR and 95% CI) ^1^ according to quintiles (Q) of different provegetarian food patterns (FP).

Provegetarian FP	Q1	Q2	Q3	Q4	Q5	p-Trend	z-Continuous ^2^
Cases/Person-years	622/29,366	494/24,015	358/17,697	429/20,395	417/21,740		2320/113,213
Age-and sex-adjusted	1 (ref)	0.97 (0.86 to 1.10)	0.93 (0.82 to 1.06)	0.94 (0.83 to 1.06)	0.77 (0.68 to 0.88)	<0.001	0.83 (0.77 to 0.90)
Repeated measures	1 (ref)	0.98 (0.87 to 1.10)	0.92 (0.81 to 1.05)	0.94 (0.83 to 1.07)	0.76 (0.67 to 0.86)	<0.001	0.82 (0.75 to 0.88)
Multivariable-adjusted ^1^	1 (ref)	0.97 (0.85 to 1.09)	0.95 (0.83 to 1.09)	0.93 (0. 82 to1.06)	0.85 (0.75 to 0.96)	0.014	0.89 (0.82 to 0.97)
Repeated measures	1 (ref)	0.98 (0.87 to 1.11)	0.94 (0.83 to 1.08)	0.94 (0. 83 to1.06)	0.83 (0.73 to 0.95)	0.005	0.88 (0.81 to 0.95)
**Healthful Provegetarian FP**							
Cases/Person-years	490/24,576	52 8/24,878	413/19,903	516/25,293	373/18,563		2320/113,213
Age-and sex-adjusted	1 (ref)	0.99 (0.87 to 1.12)	0.95 (0.84 to 1.09)	0.89 (0.79 to 1.01)	0.81 (0.71 to 0.93)	0.001	0.87 (0.80 to 0.94)
Repeated measures	1 (ref)	0.99 (0.88 to 1.12)	0.94 (0.82 to 1.07)	0.92 (0.81 to 1.04)	0.80 (0.70 to 0.92)	0.001	0.87 (0.80 to 0.94)
Multivariable-adjusted ^1^	1 (ref)	0.98 (0.86 to 1.12)	0.87 (0.76 to 1.00)	0.87 (0.76 to 0.99)	0.78 (0.67 to 0.90)	<0.001	0.84 (0.77 to 0.92)
Repeated measures	1 (ref)	0.98 (0.86 to 1.11)	0.85 (0.74 to 0.97)	0.89 (0.78 to 1.01)	0.76 (0.66 to 0.88)	<0.001	0.84 (0.77 to 0.92)
**Unhealthful Provegetarian FP**							
Cases/Person-years	544/23,243	516/24,135	514/25,004	388/20,549	358/20,282		2320/113,213
Age-and sex-adjusted	1 (ref)	0.94 (0.83 to 1.06)	0.93 (0.82 to 1.05)	0.88 (0.77 to 1.00)	0.84 (0.73 to 0.96)	0.006	0.88 (0.81 to 0.96)
Repeated measures	1 (ref)	0.95 (0.85 to 1.08)	0.92 (0.81 to 1.04)	0.87 (0.76 to 0.99)	0.86 (0.75 to 0.99)	0.012	0.88 (0.81 to 0.96)
Multivariable-adjusted ^1^	1 (ref)	1.03 (0.91 to 1.17)	1.04 (0.92 to 1.18)	1.00 (0.87 to 1.15)	1.07 (0.92 to 1.23)	0.551	1.00 (0.91 to 1.10)
Repeated measures	1 (ref)	1.04 (0.92 to 1.17)	1.03 (0.91 to 1.16)	0.99 (0.86 to 1.13)	1.09 (0.94 to 1.26)	0.416	1.01 (0.92 to 1.10)

Age was used as the underlying time variable in all the models. All the models were stratified by age groups (10-year periods) and year of recruitment (4-year periods). ^1^ Additionally adjusted for baseline body mass index (BMI, kg/m^2^, continuous), physical activity (metabolic equivalents (METs)-h/week, quartiles), hours of TV watching (quartiles), smoking status (current, never, former), marital status (single, married, other), years of university education (years, continuous), total energy intake (kcal/day, continuous), snacking between meals (yes, no), following a special diet at baseline (yes, no), parental family history of obesity (yes, no), hours of siesta (0, >0 to ≤0.5, >0.5). Robust standard errors were used. ^2^ Standardized z-scores divided by 2 (i.e., HR and 95% CI for each additional 2 z-score units).

**Table 3:** Estimates (differences and 95% confidence intervals) for average yearly weight change (g/y) according to updated quintiles (Q) of adherence to a Provegetarian Food Pattern (FP), a Healthful Provegetarian FP and an Unhealthful Provegetarian FP in the SUN Project.

	Provegetarian FP					
	Q1	Q2	Q3	Q4	Q5	p-Trend
**Absolute Yearly Weight Change (g), Adjusted Mean ^1^**		401 (343 to 460)	413 (344 to 481)	343 (280 to 405)	274 (212 to 336)	
**Age-and Sex-Adjusted Differences Versus Q1**	0 (ref)	−52 (−132 to 27)	-43 (-131 to 44)	−112 (−195 to −30)	−164 (−246 to −81)	<0.001
**Multivariable-Adjusted Differences Versus Q1 ^1^**	0 (ref)	−39 (−118 to 39)	−28 (−115 to 58)	−98 (−181 to −16)	−167 (−249 to −84)	<0.001
	**Updated Provegetarian FP (repeated measures)**					
**Absolute Yearly Weight Change (g), Adjusted Mean ^1^**	367 (321 to 413)	361 (310 to 411)	342 (282 to 402)	302 (251 to 352)	221 (168 to 274)	
**Multivariable-Adjusted Differences Versus Q1 (GEE) ^2^**	0 (ref)	−6 (−70 to 57)	−26 (−96 to 45)	−65 (−133 to 2)	−146 (−214 to -78)	<0.001
	**Healthful Provegetarian FP**					
**Absolute Yearly Weight Change (g), Adjusted Mean ^1^**	472 (413 to 531)	416 (359 to 473)	347 (283 to 411)	355 (297 to 411)	270 (203 to 338)	
**Age-and Sex-Adjusted Differences Versus Q1**	0 (ref)	−69 (−151 to 13)	−134 (−221 to −46)	−123 (−206 to −40)	−198 (−289 to −107)	<0.001
**Multivariable-adjusted differences versus Q1 ^1^**	0 (ref)	−56 (−138 to 26)	−125 (−212 to −38)	−117 (−200 to −35)	−202 (−294 to −110)	<0.001
	**Updated Healthful Provegetarian FP (repeated measures)**					
**Absolute Yearly Weight Change (g), Adjusted Mean ^1^**	416 (367 to 465)	342 (295 to 390)	285 (233 to 337)	321 (273 to 370)	216 (160 to 271)	
**Multivariable-Adjusted Differences versus Q1 (GEE) ^2^**	0 (ref)	−73 (−139 to−7)	−130 (−201 to−61)	−94 (−163 to −26)	−200 (−275 to −125)	<0.001
	**Unhealthful Provegetarian FP**					
**Absolute Yearly Weight Change (g), Adjusted Mean ^1^**	365 (306 to 425)	346 (288 to 404)	401 (343 to 458)	393 (330 to 457)	387 (320 to 455)	
**Age-and Sex-Adjusted Differences Versus Q1**	0 (ref)	−11 (−93 to 71)	50 (−32 to 133)	38 (−49 to 124)	51 (−37 to 139)	0.149
**Multivariable-Adjusted Differences Versus Q1 ^1^**	0 (ref)	−19 (−101 to 62)	35 (−47 to 118)	28 (−60 to 116)	22 (−70 to 114)	0.434
	**Updated Unhealthful Provegetarian FP (repeated measures)**					
**Absolute Yearly Weight Change (g), Adjusted Mean ^1^**	306 (256 to 355)	303 (254 to 351)	334 (287 to 381)	330 (278 to 382)	338 (283 to 393)	
**Multivariable-Adjusted Differences Versus Q1 (GEE) ^2^**	0 (ref)	−3 (−70 to 64)	28 (−39 to 96)	24 (−47 to 96)	32 (−43 to 108)	0.324

^1^ Adjusted for sex, age, baseline BMI, physical activity, hours of TV watching, smoking status, marital status, years of university education, total energy intake, snacking between meals, following a special diet at baseline, parental family history of obesity, hours of siesta sleep and year of recruitment. ^2^ GEE, generalized estimating equations for multivariable-adjusted differences (95% CIs) of yearly weight change (g/y) using repeated measures of diet after 10 years of follow-up.

**Table 4:** Risk of obesity (HR and 95% CI) ^1^ in overweight participants (n = 3935) according to quintiles of different provegetarian food patterns (FP).

Provegetarian FP	Q1	Q2	Q3	Q4	Q5	p-Trend	z-Continuous ^2^
Cases/Person-years	178/9650	162/8178	96/5655	126/7693	107/8288		2320/113,213
Age-and sex-adjusted	1 (ref)	1.06 (0.86 to 1.32)	0.93 (0.72 to 1.20)	0.90 (0.72 to 1.14)	0.71 (0.55 to 0.90)	0.003	0.78 (0.67 to 0.91)
Repeated measures	1 (ref)	1.06 (0.86 to 1.32)	0.86 (0.67 to 1.12)	0.92 (0.73 to 1.16)	0.74 (0.58 to 0.94)	0.007	0.80 (0.68 to 0.93)
Multivariable-adjusted ^1^	1 (ref)	1.09 (0.87 to 1.36)	0.91 (0.70 to 1.18)	0.96 (0.75 to 1.23)	0.79 (0.61 to 1.02)	0.056	0.82 (0.69 to 0.98)
Repeated measures	1 (ref)	1.03 (0.83 to 1.29)	0.83 (0.64 to 1.08)	0.97 (0.77 to 1.24)	0.83 (0.65 to 1.07)	0.123	0.85 (0.72 to 1.01)
**Healthful Provegetarian FP**							
Cases/Person-years	141/7423	139/7876	135/6808	149/9846	105/7510		2320/113,213
Age-and sex-adjusted	1 (ref)	0.95 (0.75 to 1.20)	1.04 (0.82 to 1.32)	0.80 (0.63 to 1.01)	0.73 (0.56 to 0.95)	0.006	0.79 (0.68 to 0.93)
Repeated measures	1 (ref)	0.98 (0.77 to 1.23)	1.05 (0.83 to 1.33)	0.78 (0.62 to 0.99)	0.74 (0.57 to 0.96)	0.004	0.79 (0.68 to 0.92)
Multivariable-adjusted ^1^	1 (ref)	0.93 (0.73 to 1.20)	1.03 (0.80 to 1.33)	0.86 (0.67 to 1.10)	0.79 (0.59 to 1.05)	0.066	0.87 (0.73 to 1.04)
Repeated measures	1 (ref)	0.94 (0.74 to 1.21)	1.04 (0.81 to 1.34)	0.82 (0.64 to 1.05)	0.79 (0.60 to 1.03)	0.036	0.86 (0.73 to 1.02)
**Unhealthful Provegetarian FP**							
Cases/Person-years	179/9930	143/8761	144/8338	108/6443	95/5991		2320/113,213
Age-and sex-adjusted	1 (ref)	0.93 (0.74 to 1.16)	0.99 (0.79 to 1.24)	0.93 (0.73 to 1.19)	0.87 (0.68 to 1.11)	0.305	0.92 (0.78 to 1.07)
Repeated measures	1 (ref)	0.92 (0.74 to 1.14)	0.97 (0.78 to 1.21)	0.90 (0.70 to 1.15)	0.86 (0.67 to 1.11)	0.256	0.92 (0.78 to 1.08)
Multivariable-adjusted ^1^	1 (ref)	1.12 (0.90 to 1.40)	1.05 (0.83 to 1.32)	1.03 (0.80 to 1.33)	0.96 (0.73 to 1.26)	0.763	0.96 (0.81 to 1.14)
Repeated measures	1 (ref)	1.12 (0.90 to 1.39)	1.04 (0.83 to 1.31)	1.02 (0.79 to 1.30)	0.95 (0.73 to 1.25)	0.698	0.97 (0.82 to 1.15)

Age was used as the underlying time variable in all the models. All the models were stratified by age groups (10-year periods) and year of recruitment (4-year periods). ^1^ Additionally adjusted for baseline BMI (kg/m^2^, continuous), physical activity (METs-h/week, quartiles), hours of TV watching (quartiles), smoking status (current, never, former), marital status (single, married, other), years of university education (years, continuous), total energy intake (kcal/day, continuous), snacking between meals (yes, no), following a special diet at baseline (yes, no), parental family history of obesity (yes, no), hours of siesta (0, >0 to ≤0.5, >0.5). Robust standard errors were used. ^2^ Standardized z-scores divided by 2 (i.e., HR and 95% CI for each additional 2 z-score units).

## Supplementary Materials

The following are available online at https://www.mdpi.com/2072-6643/11/7/1553/s1, Table S1. Food groups included in the provegetarian food patterns and food items from the SUN Project baseline FFQ, Table S2. Age-and sex-adjusted baseline characteristics (means [SDs] for continuous variables and percentages for dichotomous variables) by quintiles of the provegetarian food pattern in the SUN cohort, Table S3. Age-and sex-adjusted baseline characteristics (means [SDs] for continuous variables and percentages for dichotomous variables) by quintiles of the healthful provegetarian food pattern in the SUN cohort, Table S4. Age-and sex-adjusted baseline characteristics (means [SDs] for continuous variables and percentages for dichotomous variables) by quintiles of the unhealthful provegetarian food pattern in the SUN cohort, Table S5: Sensitivity analyses. Hazard Ratios (95% confidence intervals) of incident overweight/obesity for extreme quintiles (Q5 vs. Q1) of adherence to the different provegetarian food patterns. Figure S1. Percentage differences between extreme quintiles (quintile 1 and 5) and median scores (quintile 3) for the consumption of each food category of the provegetarian food pattern: Overall study population (The SUN Project, 1999–2015). Figure S2. Percentage differences between extreme quintiles (quintile 1 and 5) and median scores (quintile 3) for the consumption of each food category of the healthful provegetarian food pattern: Overall study population. Figure S3. Percentage differences between extreme quintiles (quintile 1 and 5) and median scores (quintile 3) for the consumption of each food category of the unhealthful provegetarian food pattern: overall study population.
