# Supplementary material for: A Provegetarian Food Pattern Emphasizing Preference for Healthy Plant-Derived Foods Reduces the Risk of Overweight/Obesity in the SUN Cohort
Source: Nutrients. 2019 Jul 9;11(7):1553. doi: 10.3390/nu11071553 (PMC6683267; doi:10.3390/nu11071553)

Table S1. Food groups included in the provegetarian food patterns and food items from the SUN cohort FFQ .

Plant Food Groups	Food items from the SUN Project FFQ
Vegetables	Swiss chard, spinach, cabbage, cauliflower, broccoli, lettuce, chicory, tomatoes, carrot, pumpkin, green beans, eggplant, zucchini, cucumber, pepper, asparagus, others
Fruits	Citrus, grapes, banana, apple, pear, strawberry, peach, apricot, nectarine, cherries, plums, figs, melon, watermelon, grapes, mango, papaya, kiwi
Legumes	Lentils, chickpeas, beans, peas
Nuts	Almonds, peanuts, hazelnuts, walnuts
Olive oil	Olive oil
Cereal grains	Breakfast cereals, white bread, white rice, pasta, whole-grain bread
Whole grains	Whole-grain bread
Refined grains	Breakfast cereals, white bread, white rice, pasta
Potatoes	Potato chips, baked potatoes
Coffee	Coffee, decaffeinated coffee
Fruit juices	Fresh orange juice, other natural fruit juices
Pastries	cookies, home-baked and ready-made cakes, muffins, donuts, croissant, cakes, churros, chocolates, nougat, marzipan
Sugary beverages	Sugar-sweetened beverages, bottled fruit or vegetable juices
**Animal Food Groups**	
Dairy	Whole milk, skim or low-fat milk, condensed milk, cream, milk shake, yogurt, custard, cheese, ice cream
Eggs	Eggs
Meat	Beef, veal, pork, lamb, liver, other viscera, chicken, turkey, serrano ham, cooked ham, spicy pork sausage, salami, mortadella, foie gras, black pudding, bacon, other cured or smoked meats, hamburger, hot dog
Fish and seafood	White fish, blue fish, salted or smoked fish, clams, mussels, shrimp, squid, octopus
Animal fat	Butter, lard
Miscellaneous food	Pizza, instant soups, mayonnaise

Table S2. Age- and sex-adjusted baseline characteristics (means [SDs] for continuous variables and percentages for dichotomous variables) by quintiles (Q) of the provegetarian food pattern in the SUN cohort.

	Provegetarian Food Pattern (n=11,554)				
	**Q1**	**Q2**	**Q3**	**Q4**	**Q5**
**N (frequency)**	2,939	2,458	1,781	2,131	2,245
**Provegetarian FP range**	13 – 32	33 – 35	36 – 37	38 – 40	41 – 55
**Provegetarian FP score**	29 (2.5)	34 (0.8)	37 (0.5)	39 (0.8)	44 (2.5)
**Age (years)**	35 (11)	35 (11)	35 (11)	35 (11)	35 (11)
**Female (%)**	73	73	73	73	74
**Unemployed (%)**	6.1	6.7	6.3	5.4	6.1
**Married (%)**	44	44	43	41	41
**Living alone (%)**	6.8	7.4	6.7	7.0	7.0
**Special diet (%)**	5.3	5.4	4.0	6.3	8.6
**Between meal snacking (%)**	35	34	33	32	31
**Smoking status (%)**					
**Never**	50	52	54	55	55
**Current**	25	23	23	21	20
**Former**	24	25	23	23	24
**Years of university education**	5.0 (1.5)	5.0 (1.4)	5.0 (1.5)	5.0 (1.5)	5.0 (1.5)
**BMI (kg/m²)**	22 (1.9)	22 (1.9)	22 (1.9)	22 (1.9)	22 (2.0)
**Family history of obesity (%)**	19	19	20	22	22
**Physical activity (METs-h/wk)**	21 (21)	22 (22)	23 (22)	24 (22)	26 (25)
**Television viewing (h/d)**	1.6 (1.2)	1.6 (1.2)	1.6 (1.2)	1.6 (1.2)	1.5 (1.2)
**Sleeping siesta (min/d)**	18 (51)	18 (48)	18 (52)	17 (44)	16 (41)
**Energy intake (kcal/d)**	2399 (577)	2317 (579)	2334 (597)	2332 (616)	2408 (631)
**Macronutrients (% E)**					
**Carbohydrates**	40 (7.0)	42 (6.5)	44 (6.6)	45 (6.6)	48 (6.7)
**CQI**	10 (2.9)	10.8 (3.1)	11 (3.1)	12 (3.1)	13 (3.1)
**Protein**	19 (3.4)	18 (3.2)	18 (2.9)	18 (2.9)	16 (2.7)
**Fat**	39 (6.2)	37 (6.0)	36 (6.3)	36 (6.5)	34 (6.5)
**SFA**	15 (3.3)	13 (2.7)	12 (2.6)	12 (2.6)	10 (2.6)
**MUFA**	16 (3.4)	16 (3.6)	16 (3.7)	16 (4.0)	15 (3.9)
**PUFA**	5.2 (1.5)	5.2 (1.6)	5.2 (1.5)	5.2 (1.6)	5.2 (1.6)
**FQI**	1.5 (0.3)	1.6 (0.3)	1.7 (0.4)	1.8 (0.4)	2.0 (0.6)
**Plant food groups[1], g/d**					
**Vegetables**	418 (272)	486 (288)	519 (312)	589 (343)	678 (355)
**Potatoes**	44 (38)	50 (40)	55 (39)	57 (41)	65 (46)
**Fruits**	258 (209)	326 (227)	371 (271)	426 (308)	487 (314)
**Fruit juices**	59 (94)	64 (94)	63 (91)	71 (95)	72 (113)
**Nuts**	4.0 (7.3)	5.8 (8.6)	7.3 (12)	8.2 (11.8)	12.3 (15)
**Legumes**	18 (15)	22 (16)	23 (20)	25 (19)	27 (18)

**Cereal grains**	79 (57)	97.6 (61.6)	106 (64)	111 (61)	129 (65)
**Whole grains**	7.1 (21)	11 (28)	13 (31)	17 (34)	22 (40)
**Refined grains**	72 (55)	87 (59)	93 (62)	95 (61)	106 (65)
**Olive oil**	14 (12)	17 (13)	19 (14)	21 (14)	24 (15)
**Pastries/sweets**	58 (44)	55 (41.7)	52 (39)	47 (36)	43 (36)
**Sugary beverages**	73 (111)	68 (99)	66 (114)	59.(88)	50 (86)
**Coffee**	65 (64)	59 (60)	61 (61)	57 (60)	54 (59)
**Animal food groups[1], g/d**					
**Dairy**	529 (264)	457 (240)	423 (222)	386 (210)	316 (202)
**Eggs**	29 (18)	25 (14)	23 (14)	21 (13)	17 (11)
**Meat**	208 (73)	184 (65)	171 (61)	160 (61)	133 (61)
**Fish and seafood**	101 (57)	96 (55)	93 (53)	94 (54)	86 (60)
**Animal fat**	1.8 (3.4)	1.3 (2.6)	1.1 (2.9)	0.8 (2.1)	0.5 (1.7)
**Miscellaneous food***	24 (36)	22 (35)	21 (36)	22 (35)	18 (32)
**Micronutrients, mg/d**					
**Vitamin C**	218 (121)	249 (139)	269 (147)	303 (160)	346 (172)
**Vitamin D**	4.0 (2.7)	3.6 (2.2)	3.6 (2.3)	3.6 (2.3)	3.4 (2.3)
**Ca**	1347 (501)	1227 (457)	1207 (443)	1188 (448)	1155 (423)
**Na**	4135 (2191)	3899 (2175)	3823 (2174)	3844 (2165)	3741 (2024)
**K**	4413 (1349)	4537 (1400)	4706 (1515)	4962 (1649)	5363 (1699)
**Mg**	388 (107)	397 (109)	411 (119)	428 (127)	463 (134)
**Folate, µg/d**	354 (151)	382 (151)	404 (167)	439 (176)	492 (189)
**Total dietary fiber (g/d)**	22 (9.4)	25 (9.5)	28 (11)	31 (12)	36 (13)
**Total alcohol intake (g/d)**	5.6 (8.7)	5.4 (7.8)	5.4 (8.3)	5.0 (6.9)	5.3 (7.5)

[1] Adjusted for energy intake using the residuals method

*Miscellaneous food: pizza, instant soups, mayonnaise.

CQI Carbohydrate Quality Index (4 – 20 index range): based on dietary fibre intake, glycemic index, whole grains:total grains ratio, solid carbohydrates: total carbohydrates ratio.

FQI Fat Quality Index (0.62 – 5.92 index range): [Monounsaturated fatty acids (MFA) + Polyunsaturated fatty acids (PUFA)] / [Saturated fatty acids (SFA) + Trans fatty acids (TFA)]

Table S3. Age- and sex-adjusted baseline characteristics (means [SDs] for continuous variables and percentages for dichotomous variables) by quintiles (Q) of the healthful provegetarian food pattern (PFP) in the SUN cohort.

	Healthful Provegetarian Food Pattern (n=11,554)				
	**Q1**	**Q2**	**Q3**	**Q4**	**Q5**
**N (frequency)**	2,460	2,543	2,022	2,582	1,947
**Healthful PFP range**	32 – 48	49 – 52	53 – 55	56 – 60	61 – 81
**Healthful PFP score**	45 (2.6)	51 (1.1)	54 (0.8)	58 (1.4)	65 (3.5)
**Age (years)**	35 (12)	35 (11)	35 (11)	35 (11)	35 (11)
**Female (%)**	72	73	73	73	73
**Unemployed (%)**	5.9	5.9	6.3	6.7	5.7
**Married (%)**	42	43	43	44	41
**Living alone (%)**	5.5	6.8	6.9	7.0	8.7
**Special diet (%)**	3.8	4.5	5.2	6.6	10.7
**Between meal snacking (%)**	37	33	32	31	30
**Smoking status (%)**					
**Never**	54	54	52	52	53
**Current**	24	23	24	23	19
**Former**	21	23	23	25	28
**Years of university education**	5.1 (1.6)	5.0 (1.5)	5.0 (1.5)	5.0 (1.5)	4.9 (1.4)
**BMI (kg/m^2)**	22 (1.9)	22 (1.9)	22 (1.9)	22 (1.9)	22 (1.9)
**Family history of obesity (%)**	19	17	21	21	25
**Physical activity (METs-h/wk)**	20 (19)	22 (21)	23 (22)	25 (24)	28 (27)
**Television viewing (h/d)**	1.6 (1.2)	1.6 (1.2)	1.6 (1.1)	1.6 (1.2)	1.5 (1.2)
**Sleeping siesta (min/d)**	20 (56)	17 (45)	17 (49)	17 (44)	16 (43)
**Energy intake (kcal/d)**	2366 (609)	2268 (595)	2304 (612)	2379 (605)	2525 (554)
**Macronutrients (% E)**					
**Carbohydrates**	43 (6.9)	43 (6.8)	44 (7.2)	44 (7.4)	46 (7.8)
**CQI**	9.0 (2.3)	10 (2.6)	11 (2.7)	12 (2.8)	15 (2.6)
**Protein**	18 (3.2)	18 (3.2)	18 (3.2)	18 (3.1)	18 (3.2)
**Fat**	38 (5.9)	37 (6.1)	37 (6.5)	37 (7.0)	35 (7.2)
**SFA**	14 (3.1)	13 (3.0)	13 (3.0)	12 (3.0)	11 (3.0)
**MUFA**	15 (3.1)	16 (3.3)	16 (3.8)	16 (4.1)	16 (4.2)
**PUFA**	5.4 (1.5)	5.2 (1.6)	5.1 (1.6)	5.1 (1.6)	5.0 (1.5)
**FQI**	1.5 (0.3)	1.6 (0.3)	1.7 (0.4)	1.8 (0.5)	2.0 (0.6)
**Plant food groups[1], g/d**					
**Vegetables**	361 (191)	461 (241)	524 (270)	601 (305)	759 (454)
**Potatoes**	65 (41)	59 (40)	54 (42)	48 (41)	39 (39)
**Fruits**	229 (155)	309 (206)	350 (230)	411 (269)	578 (395)
**Fruit juices**	64 (85)	65 (96)	65 (91)	65 (93)	66 (122)
**Nuts**	3.8 (5.6)	5.5 (7.3)	6.3 (8.5)	8.3 (12.1)	14 (18)
**Legumes**	17 (12)	21 (14)	23 (16)	25 (21)	28 (23)

**Cereal grains**	101 (63)	101 (58)	102 (62)	106 (68)	108 (72)
**Whole grains**	3.2 (13)	6.8 (17)	11 (25)	17 (35)	35 (50)
**Refined grains**	98 (62)	94 (57)	91 (60)	89 (66)	73 (63)
**Olive oil**	13 (11)	16 (11)	19 (13)	22 (15)	25 (16)
**Pastries/sweets**	61 (36)	55 (37)	53 (39)	48 (44)	37 (42)
**Sugary beverages**	86 (102)	75 (109)	67 (107)	50 (89)	33 (85)
**Coffee**	44 (54)	54 (56)	61 (61)	68 (64)	74 (65)
**Animal food groups[1], g/d**					
**Dairy**	453 (227)	442 (229)	434 (246)	415 (241)	404 (278)
**Eggs**	29 (17)	25 (15)	23 (14)	21 (13)	18 (14)
**Meat**	195 (64)	186 (66)	175 (67)	163 (67)	143 (74)
**Fish and seafood**	93 (53)	93 (50)	94 (57)	95 (55)	100 (71)
**Animal fat**	1.9 (3.3)	1.4 (2.9)	1.1 (2.6)	0.9 (2.4)	0.4 (2.0)
**Miscellaneous food***	34 (43)	24 (33)	23 (37)	17 (33)	10 (24)
**Micronutrients, mg/d**					
**Vitamin C**	196 (100)	233 (117)	266 (133)	304 (147)	395 (199)
**Vitamin D**	3.7 (2.4)	3.5 (2.1)	3.6 (2.4)	3.7 (2.6)	3.9 (2.7)
**Ca**	1173 (425)	1178 (425)	1209 (457)	1261 (481)	1384 (508)
**Na**	4555 (2614)	3884 (2065)	3874 (2225)	3703 (2109)	3508 (1627)
**K**	4164 (1203)	4384 (1292)	4630 (1406)	4997 (1479)	5961 (1851)
**Mg**	369 (99)	381 (101)	402 (110)	434 (116)	513 (135)
**Folate, µg/d**	322 (120)	363 (133)	399 (152)	448 (163)	552 (207)
**Total dietary fiber (g/d)**	21 (7.7)	24 (8.3)	27 (9.3)	31 (11)	41 (14)
**Total alcohol intake (g/d)**	4.8 (7.1)	5.1 (7.8)	5.6 (7.6)	5.7 (8.1)	5.7 (8.3)

[1] Adjusted for energy intake using the residuals method

*Miscellaneous food: pizza, instant soups, mayonnaise.

CQI Carbohydrate Quality Index (4 – 20 index range): based on dietary fibre intake, glycemic index, whole grains:total grains ratio, solid carbohydrates: total carbohydrates ratio.

FQI Fat Quality Index (0.62 – 5.92 index range): [Monounsaturated fatty acids (MFA) + Polyunsaturated fatty acids (PUFA)] / [Saturated fatty acids (SFA) + Trans fatty acids (TFA)]

Table S4. Age- and sex-adjusted baseline characteristics (means [SDs] for continuous variables and percentages for dichotomous variables) by quintiles (Q) of the unhealthful provegetarian food pattern (PFP) in the SUN cohort.

	Unhealthful Provegetarian Food Pattern (n=11,554)				
	**Q1**	**Q2**	**Q3**	**Q4**	**Q5**
**N (frequency)**	2,492	2,508	2,525	2,053	1,976
**Unhealthful PFP range**	28 – 48	49 – 52	53 – 56	57 – 60	61 – 80
**Unhealthful PFP score**	45 (2.8)	51 (1.1)	55 (1.1)	58 (1.1)	64 (3.2)
**Age (years)**	35 (11)	35 (11)	35 (11)	35 (11)	35 (11)
**Female (%)**	73	73	73	73	73
**Unemployed (%)**	7.2	5.7	5.9	6.6	5.7
**Married (%)**	44	43	42	43	42
**Living alone (%)**	7.9	7.1	8.1	6.	5.2
**Special diet (%)**	9.6	7.1	5.0	3.6	3.3
**Between meal snacking (%)**	27	31	33	34	40
**Smoking status (%)**					
**Never**	51	52	53	55	55
**Current**	20	24	23	22	24
**Former**	28	23	23	22	21
**Years of university education**	5.0 (1.4)	5.0 (1.5)	5.0 (1.5)	5.0 (1.5)	5.0 (1.5)
**BMI (kg/m^2)**	22 (1.9)	22 (1.9)	22 (1.9)	22 (1.9)	22 (1.9)
**Family history of obesity (%)**	23	20	21	19	18
**Physical activity (METs-h/wk)**	27 (25)	24 (23)	23 (23)	22 (20)	21 (20)
**Television viewing (h/d)**	1.5 (1.1)	1.6 (1.2)	1.6 (1.2)	1.6 (1.2)	1.6 (1.3)
**Sleeping siesta (min/d)**	16 (40)	18 (51)	18 (50)	17 (44)	19 (54)
**Energy intake (kcal/d)**	2195 (545)	2220 (589)	2331 (582)	2469 (586)	2706 (549)
**Macronutrients (% E)**					
**Carbohydrates**	41 (7.0)	43 (7.2)	44 (7.1)	45 (6.8)	47 (7.1)
**CQI**	13 (3.1)	12 (3.1)	11 (3.0)	11 (3.0)	9.5 (2.6)
**Protein**	20 (2.9)	19 (2.9)	18 (2.8)	17 (2.4)	15 (2.7)
**Fat**	37 (6.5)	37 (6.8)	37 (6.5)	36 (6.3)	36 (6.8)
**SFA**	12 (3.3)	13 (3.3)	13 (3.2)	13 (3.1)	13 (3.0)
**MUFA**	16 (3.7)	16 (3.8)	16 (3.7)	16 (3.5)	15 (3.9)
**PUFA**	4.9 (1.2)	5.0 (1.4)	5.2 (1.6)	5.3 (1.6)	5.6 (2.0)
**FQI**	1.8 (0.5)	1.7 (0.5)	1.7 (0.5)	1.7 (0.4)	1.7 (0.5)
**Plant food groups[1], g/d**					
**Vegetables**	692 (329)	586 (342)	517 (291)	450 (290)	358 (284)
**Potatoes**	39 (29)	49 (35)	54 (39)	61 (44)	70 (55)
**Fruits**	458 (271)	396 (266)	358 (279)	333 (273)	265 (277)
**Fruit juices**	59 (84)	62 (88)	70 (101)	69 (104)	69 (113)
**Nuts**	9.6 (13)	8.2 (12)	7.6 (12)	5.9 (10)	4.6 (10)
**Legumes**	27 (18)	24 (16)	23 (17)	21 (18)	18 (18)

**Cereal grains**	89 (48)	99 (57)	102 (60)	108 (69)	123 (84)
**Whole grains**	23 (36)	17 (35)	13 (30)	7.9 (25)	3.4 (21)
**Refined grains**	65 (40)	17 (14)	89 (57)	100 (66)	120 (82)
**Olive oil**	22 (13)	20 (14)	19 (14)	17 (14)	15 (16)
**Pastries/sweets**	36 (24)	43 (28)	50 (34)	59 (43)	75 (58)
**Sugary beverages**	37 (60)	54 (82)	63 (90)	74 (106)	99 (149)
**Coffee**	75 (63)	64 (59)	58 (60)	51 (58)	45 (56)
**Animal food groups[1], g/d**					
**Dairy**	496 (250)	451 (243)	433 (240)	392 (228)	346 (225)
**Eggs**	26 (13)	24 (15)	23 (15)	22 (15)	20 (16)
**Meat**	186 (66)	177 (68)	175 (71)	169 (69)	154 (71)
**Fish and seafood**	118 (53)	103 (54)	92 (56)	83 (55)	67 (45)
**Animal fat**	1.2 (2.4)	1.3 (3.0)	1.1 (2.6)	1.2 (2.8)	0.9 (2.9)
**Miscellaneous food***	23 (30)	23 (34)	22 (36)	22 (39)	18 (38)
**Micronutrients, mg/d**					
**Vitamin C**	322 (158)	281 (157)	266 (149)	256 (144)	233 (150)
**Vitamin D**	4.2 (2.6)	3.7 (2.4)	3.6 (2.6)	3.4 (2.2)	3.1 (2.0)
**Ca**	1330 (463)	1231 (485)	1225 (462)	1188 (443)	1174 (435)
**Na**	3662 (1891)	3745 (2136)	3887 (2166)	4076 (2365)	4301 (2222)
**K**	5190 (1562)	4761 (1627)	4685 (1535)	4630 (1499)	4578 (1493)
**Mg**	440 (124)	410 (130)	408 (121)	408 (117)	414 (113)
**Folate, μg/d**	473 (170)	421 (183)	402 (166)	384 (157)	360 (167)
**Total dietary fiber (g/d)**	32 (12)	29 (13)	27 (12)	27 (11)	26 (12)
**Total alcohol intake (g/d)**	5.2 (7.1)	5.1 (7.1)	5.5 (8.4)	5.6 (8.0)	5.5 (8.5)

[1] Adjusted for energy intake using the residuals method

*Miscellaneous food: pizza, instant soups, mayonnaise.

CQI Carbohydrate Quality Index (4 – 20 index range): based on dietary fibre intake, glycemic index, whole grains:total grains ratio, solid carbohydrates: total carbohydrates ratio.

FQI Fat Quality Index (0.62 – 5.92 index range): [Monounsaturated fatty acids (MFA) + Polyunsaturated fatty acids (PUFA)] / [Saturated fatty acids (SFA) + Trans fatty acids (TFA)]

Table S5. Sensitivity Analyses. Hazard Ratios (95% confidence intervals) of incident overweight/obesity for extreme quintiles (Q5 vs. Q1) of adherence to the different provegetarian food patterns.

			Provegetarian		*Healthful*		*Unhealthful*	
	**Cases**	**N**	**Q5 vs. Q1 (ref)**	***p* trend**	**Q5 vs. Q1 (ref)**	***p* trend**	**Q5 vs. Q1 (ref)**	***p* trend**
Overall	2320	11,554	0.85 (0.75, 0.96)	0.014	0.78 (0.67, 0.90)	<0.001	1.07 (0.92, 1.23)	0.551
Energy limits: Percentiles 5-95	2301	11,637	0.82 (0.72, 0.94)	0.005	0.76 (0.66, 0.89)	<0.001	1.04 (0.89, 1.21)	0.633
Excluding participants with no answer in >12 items out of 136 in the baseline FFQ	2116	10,613	0.90 (0.79, 1.04)	0.094	0.82 (0.70, 0.95)	0.002	1.02 (0.87, 1.19)	0.910
Including participants with weight change >10kg over the past 5 years before entering the study	2449	11,874	0.87 (0.76, 0.98)	0.023	0.79 (0.69, 0.91)	0.001	1.02 (0.88, 1.18)	0.824
Additionally adjusted for weight gain ≥3kg over the past 5 years before entering the cohort	2320	11,554	0.85 (0.75, 0.97)	0.014	0.78 (0.67, 0.90)	<0.001	1.06 (0.92, 1.23)	0.565
Excluding participants with BMI>24.5 kg/m² at baseline	1739	10,754	0.79 (0.68, 0.92)	0.002	0.74 (0.62, 0.87)	<0.001	0.99 (0.83, 1.17)	0.915
Excluding early cases of overweight/obesity (first 2 y)	1547	10,780	0.81 (0.69, 0.95)	0.005	0.75 (0.63, 0.90)	0.001	0.96 (0.80, 1.15)	0.901
Considering obesity as outcome (BMI ≥ 30 kg/m²)	759	15,489	0.79 (0.62, 1.01)	0.051	0.78 (0.60, 1.02)	0.037	0.97 (0.75, 1.25)	0.800
Truncating follow-up at 10 years	1884	11,554	0.85 (0.74, 0.98)	0.030	0.74 (0.63, 0.86)	<0.001	1.15 (0.98, 1.35)	0.227

Age was the underlying time variable in all models.
Adjusted for sex, baseline BMI, physical activity, hours of TV watching, smoking status, marital status, years of university education, total energy intake, snacking between meals, following a special diet at baseline, parental family history of obesity, hours of siesta. Stratified by age groups and year of recruitment.

Figure S1. Percentage differences[1] between extreme quintiles (quintile 1 and 5) and median scores (quintile 3) for the consumption of each food category of the provegetarian food pattern: overall study population (The SUN Project, 1999-2015).

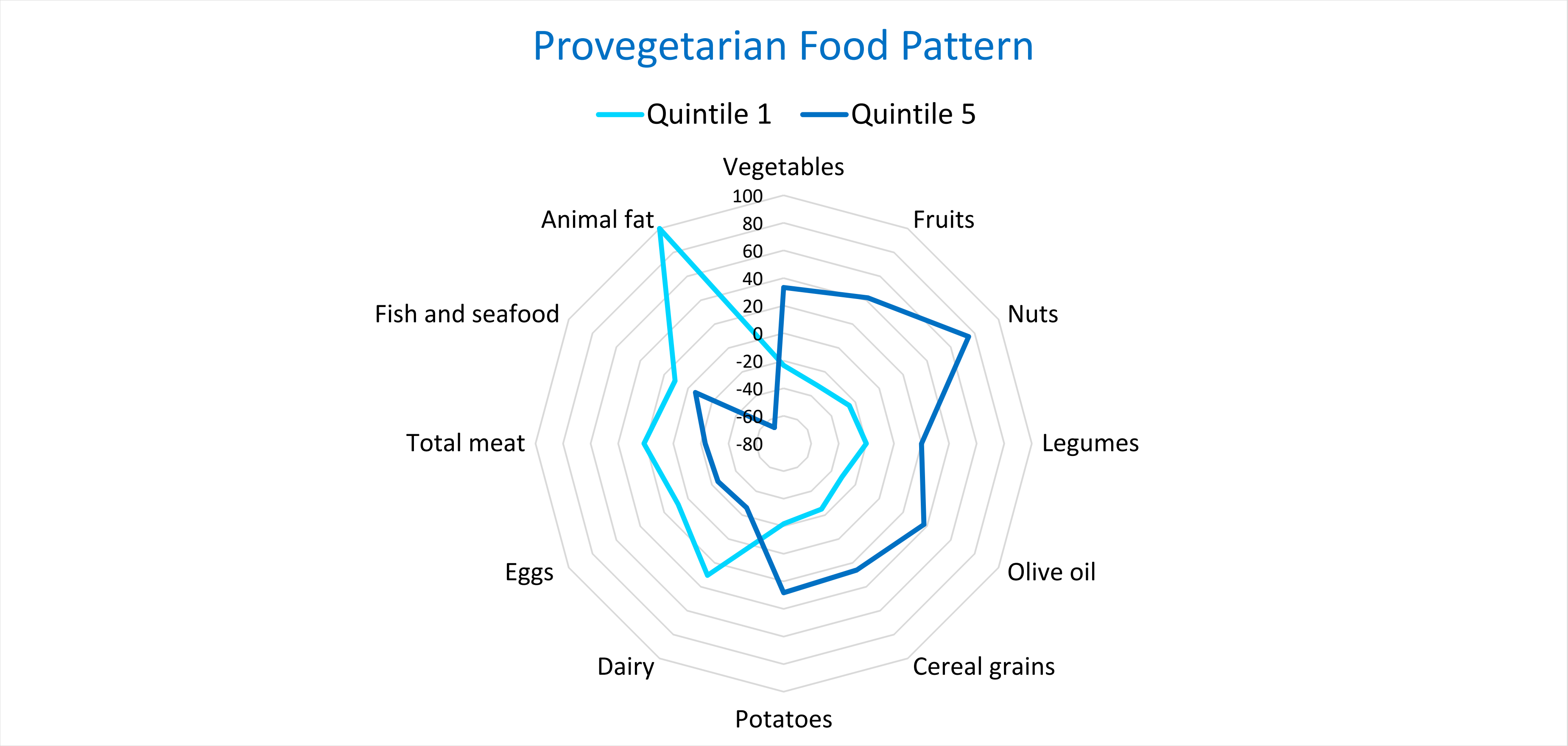

[1] For example, the median vegetable intake (g/day) in the provegetarian score was 467 in the overall study population. The vegetable intake in the lowest provegetarian quintile was 358 (77% of median intake), whereas the vegetable intake in the highest provegetarian quintile was 622 (133% of median intake). We conceptualized the median as 100% and plotted the percent differences from the median.

Figure S2. Percentage differences between extreme quintiles (quintile 1 and 5) and median scores (quintile 3) for the consumption of each food category of the healthful provegetarian food pattern: overall study population (The SUN Project, 1999-2015).

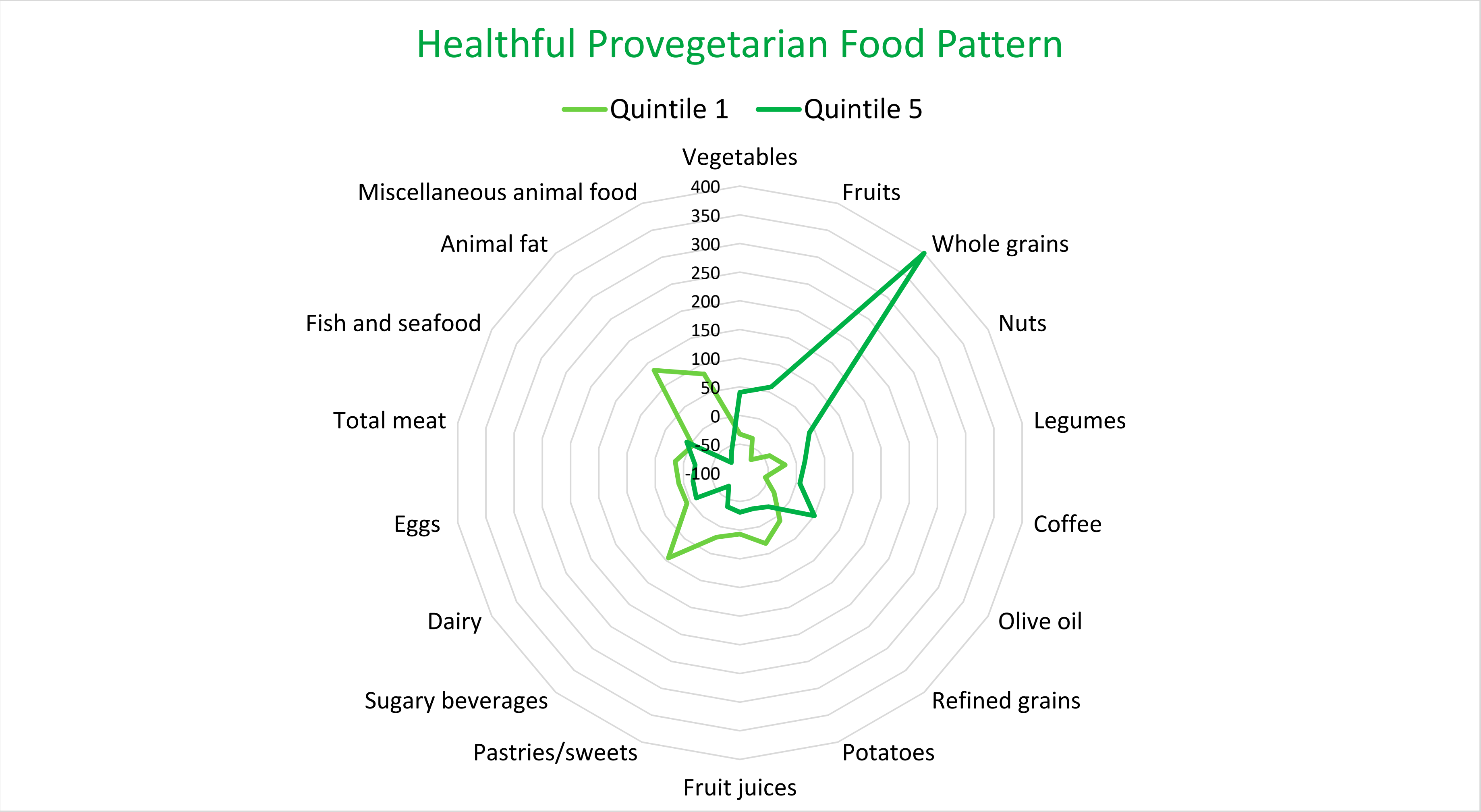

Figure S3. Percentage differences between extreme quintiles (quintile 1 and 5) and median scores (quintile 3) for the consumption of each food category of the unhealthful provegetarian food pattern: overall study population (The SUN Project, 1999-2015).

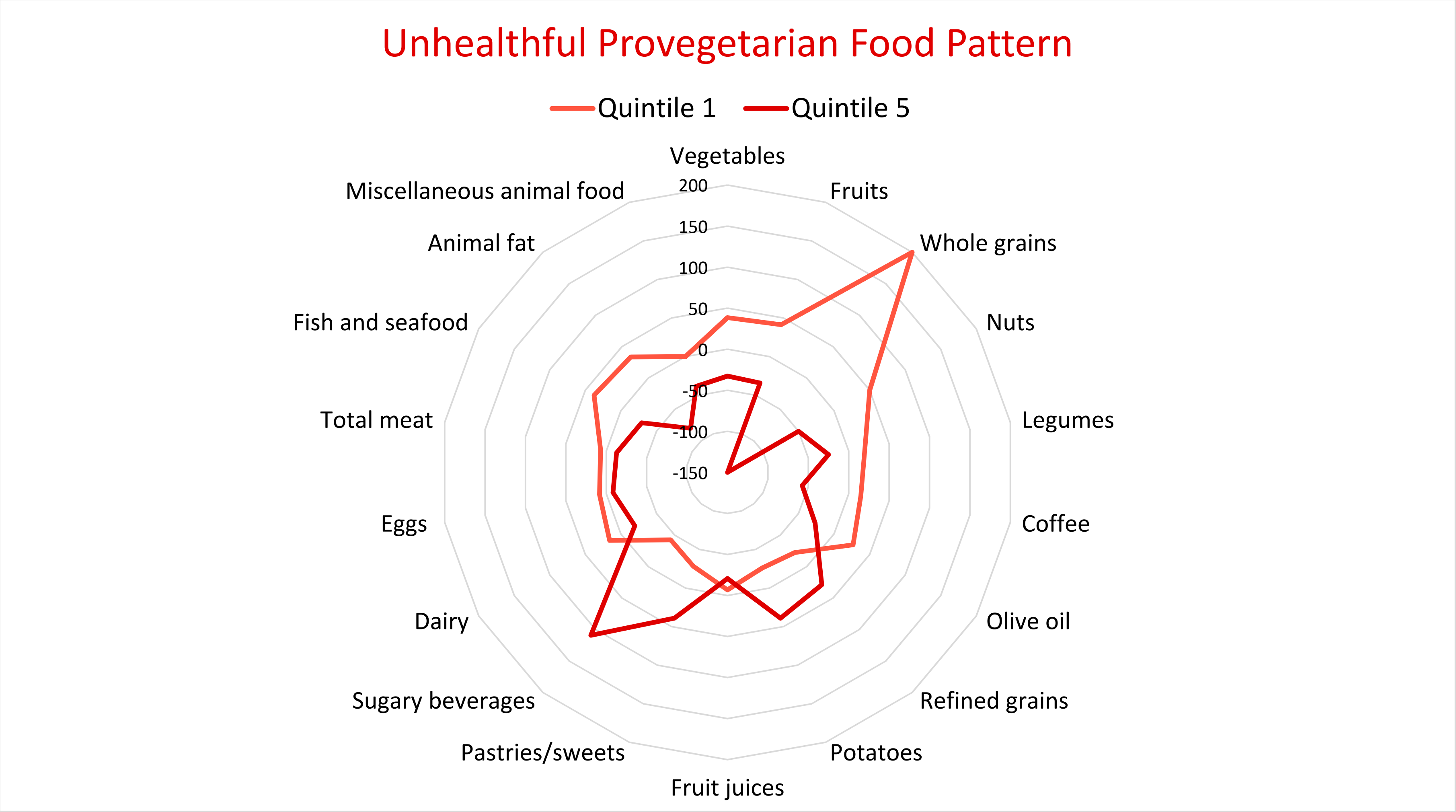

Supplement: Supplementary file 1 [file nutrients-11-01553-s001.pdf]
